# Development of a variety and quality evaluation method for Amomi fructus using GC, electronic tongue, and electronic nose

**DOI:** 10.3389/fchem.2023.1188219

**Published:** 2023-06-15

**Authors:** Fuguo Hou, Xuehua Fan, Xinjing Gui, Han Li, Haiyang Li, Yanli Wang, Junhan Shi, Lu Zhang, Jing Yao, Xuelin Li, Ruixin Liu

**Affiliations:** ^1^ School of Pharmacy, Henan University of Chinese Medicine, Zhengzhou, China; ^2^ Department of Pharmacy, The First Affiliated Hospital of Henan University of Chinese Medicine, Zhengzhou, China; ^3^ Henan Province Engineering Research Center for Clinical Application, Evaluation and Transformation of Traditional Chinese Medicine, Zhengzhou, China; ^4^ Co-Construction Collaborative Innovation Center for Chinese Medicine and Respiratory Diseases by Henan & Education Ministry of China, Henan University of Chinese Medicine, Zhengzhou, China; ^5^ Henan Key Laboratory for Clinical Pharmacy of Traditional Chinese Medicine, Zhengzhou, China; ^6^ Engineering Research Center for Pharmaceutics of Chinese Materia Medica and New Drug Development, Ministry of Education, Beijing, China

**Keywords:** variety and quality evaluation, bionic sensory, volatile components, qualitative and quantitative, predictive model, amomi fructus (AF)

## Abstract

*Amomi fructus* is rich in volatile components and valuable as a medicine and edible spice. However, the quality of commercially available *A. fructus* varies, and issues with mixed sources and adulteration by similar products are common. In addition, due to incomplete identification methods, rapid detection of the purchased *A. fructus* quality is still an issue. In this study, we developed qualitative and quantitative evaluation models to assess the variety and quality of *A. fructus* using GC, electronic tongue, and electronic nose to provide a rapid and accurate variety and quality evaluation method of *A. fructus*. The models performed well; the qualitative authenticity model had an accuracy of 1.00 (n = 64), the accuracy of the qualitative origin model was 0.86 (n = 44), and the quantitative model was optimal on the sensory fusion data from the electronic tongue and electronic nose combined with borneol acetate content, with *R* ^2^ = 0.7944, RMSEF = 0.1050, and RMSEP = 0.1349. The electronic tongue and electronic nose combined with GC quickly and accurately evaluated the variety and quality of *A. fructus*, and the introduction of multi-source information fusion technology improved the model prediction accuracy. This study provides a useful tool for quality evaluation of medicine and food.

## Graphical Abstract

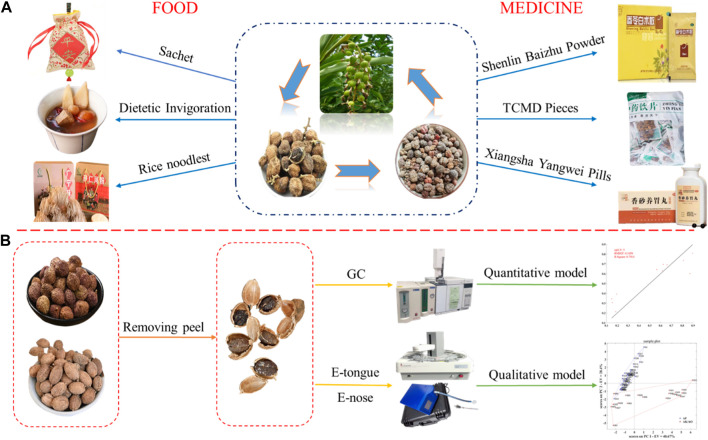

## 1 Introduction

*Amomi fructus*, also known as Sharen in China, is the dried fruit of *Amomum villosum* Lour. and *A. villosum* Lour. var.*xanthioides* T.L.Wu et Senjen and *Amomum longiligulare* T.L.Wu (Chinese Pharmacopoeia Commission, 2020). The fruits are harvested in summer and autumn when they are ripe and dried in the Sun or at low temperatures using air blast drying box. *Amomum villosum* is a tropical plant that prefers the warm and humid climate of the southern subtropical monsoon rainforest and is grown in Guangdong, Yunnan and Hainan provinces in China and in Myanmar, Vietnam, and Thailand.

*Amomi fructus* contains rich volatile oil components including borneol acetate, camphor, borneol, camphene, α-pinene, and β-pinene (Chen et al., 2021; Huang et al., 2021). These components have applications in the pharmaceutical and food industries, earning the fruit the name “medicine food homology”. *Amomi fructus* has been used for thousands of years as an important traditional Chinese medicine with a wide range of pharmacological effects such as anti-ulcer (Liu et al., 2022), anti-inflammatory, analgesic, anti-diarrheal and anti-bacterial (Li et al., 2015). It has significant effects in the treatment of diseases such as functional digestive disorders, gastritis, *Helicobacter pylori* infection in children, and acute lung injury (Suo et al., 2018; Zhao et al., 2022) and it has significant potential for scientific research and new drug development. It is used in proprietary Chinese medicines such as the Xiangsha Yangwei Pill, Jianpi Pill, and Shenling Baizhu Granules (Zhang et al., 2020; Zu et al., 2022), and directly used when mashed in the form of a traditional Chinese medicine decoction (Nie et al., 2018). *Amomi fructus* is also a food and can be used as a spice which is finely ground into a sachet or aromatherapy. The complete fruit or seed can be used as a seasoning, with common *A. fructus* meals including *A. fructus* stew ribs, *A. fructus* crucian soup (Yu and Gao, 2017), and *A. fructus* porridge (Xue, 2019; Zhao, 2020), *etc.* In addition, a series of *A. fructus* by-products such as *A. fructus* wine, *A. fructus* honey, and *A. fructus* rice noodles have been developed in recent years.

The variety and quality of commercially available *A. fructus* vary greatly due in part to the easy mixing of sources and doping of similar varieties (Hou et al., 2015). Due to the lack of identification of some samples sold and incomplete identification methods, the quality of commercial *A. fructus* is uncertain. The traditional identification methods of *A. fructus*, include tasting, sniffing, and visual inspection (Hou et al., 2015), and although the identification speed is fast, the resulting description is relatively vague, subjective, and lacks objective quantitative criteria, which limits its practical applications. Modern identification methods include GC (Xu et al., 2018), GC-MS(Wang et al., 2021a; Gu et al., 2022), and DNA barcoding (Huang et al., 2017; Lu et al., 2021) which have accurate and reliable results; however, the sample pretreatment is cumbersome, time-consuming and has high technical operational requirements. Therefore, a rapid and accurate quality identification method for *A. fructus* is urgently needed (Gui et al., 2023).

Bionic sensory technologies such as e-tongue and e-nose (Weng et al., 2022; Gui et al., 2023), can be used to objectify the trait characteristics of medicine or food. The integration of bionic sensory and modern analysis instrumental yields the advantages of both “fast” sensory response and “quantitative” instrumental analysis, affording fast analysis, high sensitivity, strong reliability, good repeatability, and strong integrity (Xie et al., 2016). Multi-source Information Fusion (MIF) technology was first used by the U.S. Navy in the military field, and has become an emerging multidisciplinary approach that can combine and optimize data from multiple bionic sensory sources to obtain more detailed and accurate reasoning than an individual source, and it is now being used in the quality evaluation of medicine and food (Dai et al., 2018; Xu et al., 2019; Lan et al., 2020; Jiang et al., 2021; Jing et al., 2022).

This study aims to qualitatively and quantitatively evaluate the variety and quality of *A. fructus* using the combination of GC, e-tongue, and e-nose to establish a rapid and accurate method to evaluate the variety and quality of *A. fructus* and to provide a reference for the quality evaluation of other foods and medicines.

## 2 Materials and methods

### 2.1 Drugs and reagents

A total of 44 batches of *A. fructus* from four origins were collected in this study, including AF-1 for Yunnan *A. fructus* from China (S1-S21), AF-2 for Guangdong *A. fructus* from China (S22-S34), AF-3 for Hainan *A. fructus* from China (S35-S39), and AF-4 for Myanmar *A. fructus* (S40-S44). In addition, 10 batches of each of *Alpiniae katsumadai semen* (AK) and *Alpiniae oxyphyllae fructus* (AO) were collected as counterfeit of *A. fructus*, with AK for S45-S54 and AO for S55-S64.

Camphor, borneol, and borneol acetate were purchased from Shanghai Yuanye Bio-Technology Co., Ltd. (Shanghai, China). Anhydrous ethanol (analytical purity, Tianjin Yongda Chemical Reagent Co., Ltd.); tartaric acid, potassium chloride (reference solution); potassium chloride, pure water, potassium hydroxide (positive cleaning solution); anhydrous ethanol, concentrated hydrochloric acid (negative cleaning solution).

### 2.2 The percentage of peel analysis

Appropriate amounts of each batch of *A. fructus* samples were taken, their peels and seeds were separated and weighed, and the peel percentage by weight was calculated and recorded.

### 2.3 Volatile component content analysis

Control solutions of camphor, borneol, and borneol acetate at concentrations of 0.942 mg mL^-1^, 0.870 mg mL^-1^, and 2.000 μL mL^-1^, respectively, were made through dissolving and dilution by anhydrous ethanol.

A 1 g sample of *A. fructus* seed/peel powder was added to a conical flask with 25 mL anhydrous ethanol and extracted with ultrasonic waves at 40 kHz for 30 min. After cooling to room temperature, the processed samples were shaken well and centrifuged at 5,000 r·min^-1^ for 15 min. Finally, the solution was filtered using a 0.45 μm filter membrane to obtain the seed/peel sample solution.

The GC system included an Agilent 7890A instrument (Agilent Technologies Co., Ltd. United States of America) with a flame ionization detector (FID): GC conditions: Agilent HP-5 column (0.25 μm, 0.32 mm × 30 m). Nitrogen was used as the carrier gas at a constant flow rate of 1.1 mL/min. The column was heated to 63 C, then increased at a rate of 5°C/min to 130°C and held for 5 min. The temperature was then further increased at a rate of 20 C/min to 230°C and held for 5 min. The column oven temperature was 300°C and the injection volume was 1 μL.

### 2.4 Bionic sensory analysis

#### 2.4.1 Measurement of bionic taste data by e-tongue

To prepare the positive electrode cleaning solution, 7.46 g of potassium chloride was accurately weighed and dissolved with 500 mL of distilled water. Then 300 mL of absolute ethanol solution and 0.56 g of potassium hydroxide were added while stirring. After dissolving, the solution was transferred to a 1,000 mL volumetric flask and the volume was adjusted to 1,000 mL.

The negative electrode cleaning solution was prepared by mixing 300 mL of absolute ethanol with 500 mL of distilled water by shaking, then mixing in 8.3 mL of concentrated hydrochloric acid followed by transferring to a 1,000 mL volumetric flask and adjusting the volume to 1,000 mL.

The glass electrode immersion maintenance solution was prepared by adding 248.2 g potassium chloride in 900 mL distilled water, dissolving it completely by ultrasound, and adjusting the volume to 1,000 mL.

The reference solution was prepared by first weighing 0.18 g tartaric acid and 8.946 g potassium chloride in a 1 L volumetric flask, then adding distilled water to fix the volume. Next, the solution was shaken and poured into a 4 L container. Finally, add 3 L of distilled water to the 4 L container with the volumetric flask.

The sample was prepared by taking 3 g of powder sample, adding 100 mL of reference solution to dissolve, shaking for 10 min, sonicating for 10 min, filtering through, then placing in a special beaker (about 35 mL) for measurement by the e-tongue.

The SA402 B e-tongue (Intelligent Sensor Technology, Inc., Japan) was used to test the bionic taste data of each sample. The e-tongue included eight sensors: C00, AE1, CA0, CT0, AAE, AN0, BT0 and GL1 (Table 1). A total of 11 taste information values are output from 8 sensors, each representing a different type of taste information, and the difference between the different taste information is indicated by the level of the response value. The type of measurement adopted in e-tongue is potentiometric measurement. First, the sensors were cleaned for 90 s in a cleaning solution, followed by 120 s with a reference solution, and continued with another reference solution for 120s. The sensors were zeroed in the equilibrium position for 30 s. After reaching the equilibrium condition, the test started with a test time of 30 s, and the sensors outputted the first taste value. Then, the sensors were briefly cleaned in the two groups of reference solutions for 3 s, and the sensors were inserted into the new reference solution to test the aftertaste for 30 s. It was cycled 4 times. The result of the first cycle was removed, and the average value of the last 3 times was considered the test result. Among them, the sweet taste sensor GL1 was tested for 5 times, and the first cycle and the last cycle were removed, and the average data of the middle three times were taken as the test results. Each cleaning, equilibrating, and testing of the sensors were performed in different sample cups (Liu et al., 2014; Li et al., 2023). The total test time per sample was approximately 90 min when all types of sensors were used.

**TABLE 1:** Sensors information.

Sensor attribution	Sensor name	Sensor description and sensitivities
E-tongue	C00	Bitterness, aftertaste-B
	AE1	Astringency, aftertaste-A
	CA0	Sourness
	CT0	Saltiness
	AAE	Umami, richness
	AN0	B-bitterness2
	BT0	H-bitterness
	GL1	Sweetness
E-nose	W1C	Aromatic organic compounds
	W5S	Nitrogen oxides, negative signal
	W3C	Ammonia, aromatic compounds
	W6S	Hydrogen gas
	W5C	Alkanes, aromatic compounds, and non-polar organic compounds
	W1S	Methane, broad range of organic compounds
	W1W	Inorganic sulfur compounds, terpenes and sulfur containing organic compounds
	W2S	Alcohol, aromatic compounds
	W2W	Aromatic compounds, inorganic sulfur and organic compounds
	W3S	High concentrations of methane and aliphatic organic compounds

#### 2.4.2 Measurement of bionic olfactory data by e-nose

The PEN3.5 e-nose (AIRSENSE Analytics GmbH, Germany) included ten sensors: W1C, W5S, W3C, W6S, W5C, W1S, W1W, W2S, W2W, W3S (Table 1). Different sensors can detect different types of chemicals and indicate the level of compound content by the level of response value. The type of measurement adopted in e-nose is resistance measurement. The sample was weighed 3 g and placed in a 50 mL centrifuge tube and sealed with a sealing film. The sample odor information was collected by static headspace sampling method and the headspace generation time was 30 min.

After connecting the PEN3.5 e-nose to the computer, we ran its supporting software and set the e-nose parameters for each test, including sampling and cleaning time. We selected a folder and path to save the results and the name of each sample to be tested and started the test. The sample inlet flow rate was set to 400 mL and the sampling time was 100 s. Three parallel measurements were taken, and the data at 80 s inlet time was taken as the results. The experiment was conducted at room temperature of about 24 C and relative humidity of about 82%. The total time of measurements for each sample is about 3 min.

### 2.5 Statistical analysis

A comparative analysis of the peel and seed samples was conducted using Origin 2022. Principal Component Analysis (PCA) was conducted using SIMCA 14.1, which is one of the most frequently used unsupervised chemometric tools. It allowed the projection of data from a higher to a lower-dimensional space so that it simplifies the interpretation of variables between the samples (Barbosa et al., 2020). Orthogonal Partial Least Square Discriminant Analysis (OPLS-DA) (Wang et al., 2021b) was conducted using SIMCA 14.1, Principal Component Analysis Discriminant Analysis (PCA-DA) and Partial Least Squares Discriminant Analysis (PLS-DA) were conducted using the MATLAB R2016B software, which a supervised discriminant analysis statistical method, and it is very beneficial to finding relevant information related with particular samples and variables of a dataset (Zhang et al., 2022). Partial Least Squares Regression (PLSR) (Htet et al., 2021) was also conducted using the MATLAB R2016B software, which is to find a linear regression model by projecting predictive variables and observed variables into a new space.

## 3 Results and discussion

### 3.1 The percentage of peel analysis

**FIGURE 1:**
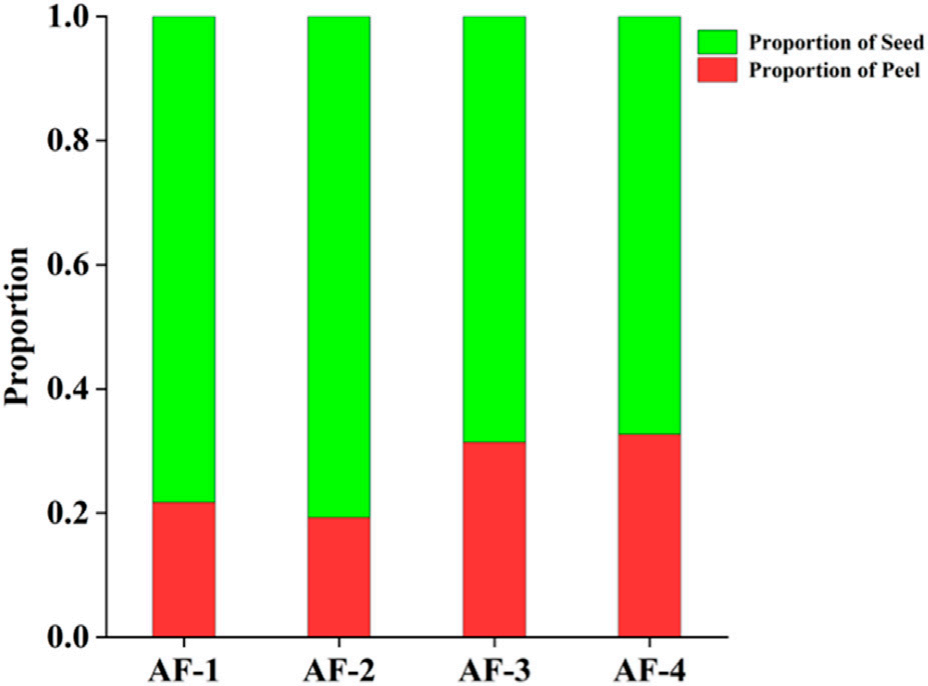
The percentage of peel in Yunnan, Guangdong, Hainan and Myanmar *Amomi fructus*.

The percentage of peel results from the Yunnan, Guangdong, Hainan, and Myanmar *A. fructus* are reported in Figure 1. The peel percentage of *A. fructus* from Yunnan and Guangdong were around 0.2, while the peel percentage of Hainan and Myanmar *A. fructus* was around 0.3, which represented a significant difference (*p* < 0.05). Compared to Yunnan and Guangdong *A. fructus*, the peels of Hainan and Myanmar *A. fructus* are shrunken and thicker, consistent with the measurement results.

### 3.2 Volatile component content analysis

#### 3.2.1 Establishment and analysis of GC fingerprints

**FIGURE 2:**
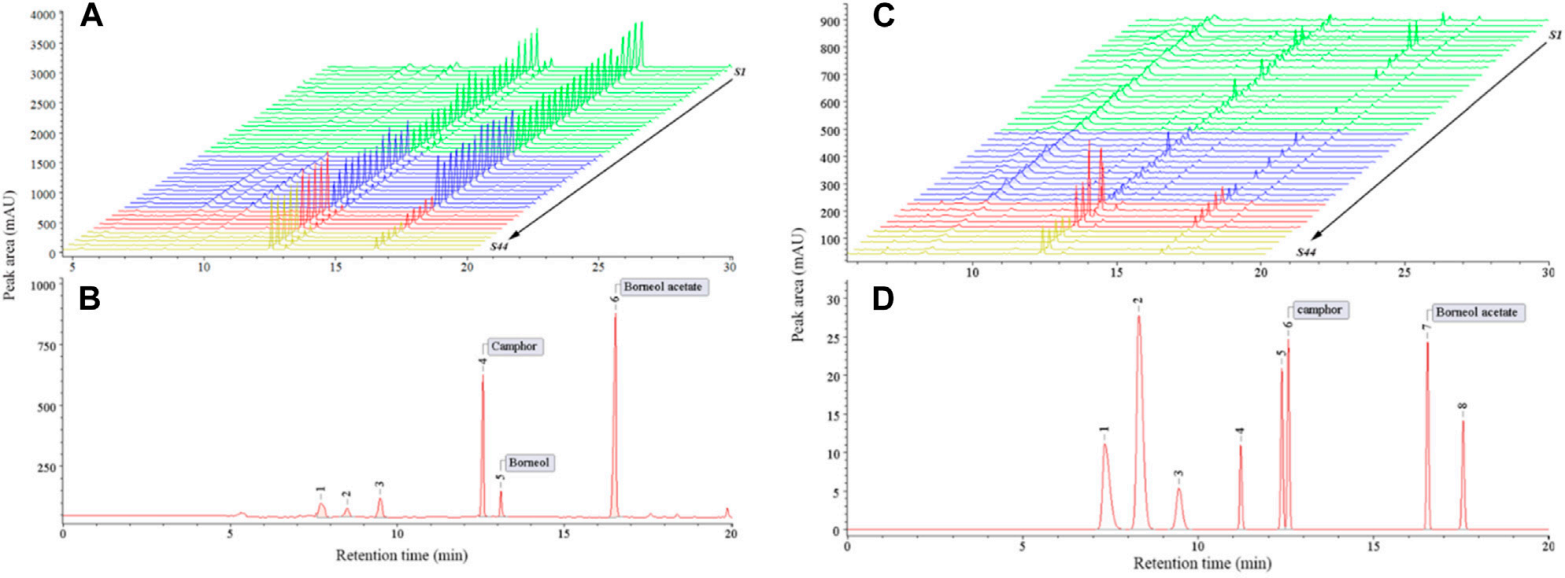
GC fingerprints of 44 batches of *Amomi fructus* seed **(A)** and peel **(C)**, common mode characteristic chromatogram of seed **(B)** and peel **(D)**.

The GC fingerprints and common patterns of 44 batches of *A. fructus* seeds and peels are illustrated in Figure 2. A total of 6 common peaks were extracted from the seed fingerprints. Among these peaks, 3 common peaks were identified by comparison to the reference fingerprint, with peak 4 being camphor, peak 5 being borneol, and peak 6 being borneol acetate. A total of 8 common peaks were extracted from the peel fingerprints, two of which, peak 6 (camphor) and peak 7 (borneol acetate), were identified by comparison to the reference fingerprint. The fingerprints of seeds and peels were analyzed for similarity analysis with their common patterns, revealing that in both seeds and peels the fingerprints of Yunnan and Guangdong *A. fructus* significantly differed from those of Hainan and Myanmar (*p* < 0.01). The average fingerprint similarity was above 0.9 for Yunnan and Guangdong *A. fructus* and below 0.7 for Hainan and Myanmar *A. fructus.*

#### 3.2.2 Comparative analysis of the content of volatile components of seeds and peels

**FIGURE 3:**
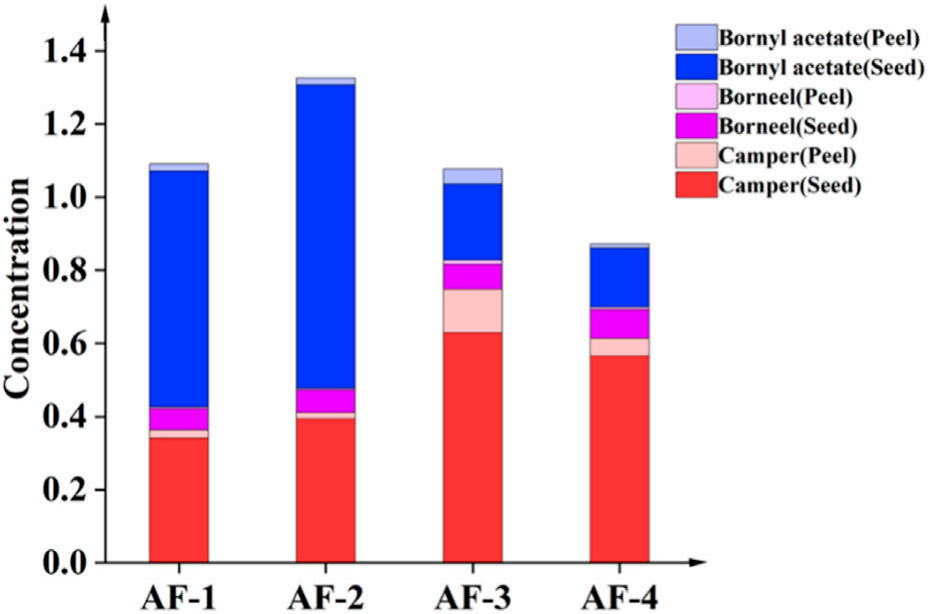
Contents of volatile components in *Amomi fructus* seed and peel.

The volatile component content in seeds was significantly higher than in peels (*p* < 0.01) (Figure 3). The content of borneol acetate in the seeds of the Guangdong *A. fructus* was significantly higher than in the *A. fructus* from Yunnan, Hainan and Myanmar (*p* < 0.05), and the content of borneol acetate in the Yunnan *A. fructus* seeds was higher than in those from Hainan and Myanmar (*p* < 0.01). There was no significant difference in seed-borneol content across the four origins (*p* > 0.05). The camphor content in the seeds from Hainan and Myanmar was significantly higher than those from Yunnan and Guangdong (*p* < 0.01). The content of all three volatile components in the peels was higher in the sample from Yunnan than from Hainan (*p* < 0.05), and the peel camphor and borneol contents were higher in the Guangdong sample than in the Hainan sample (*p* < 0.01).

Given the differences in the edible and medicinal parts of *A. fructus*, in some cases, the intact fruits are used directly, and in other cases, only the seeds are used after peeling. When using different parts and varieties of *A. fructus*, the component content of seeds and peels as well as peel percentage should be taken into account for dosage calculations.

#### 3.2.3 OPLS-DA of the seed components

**FIGURE 4:**
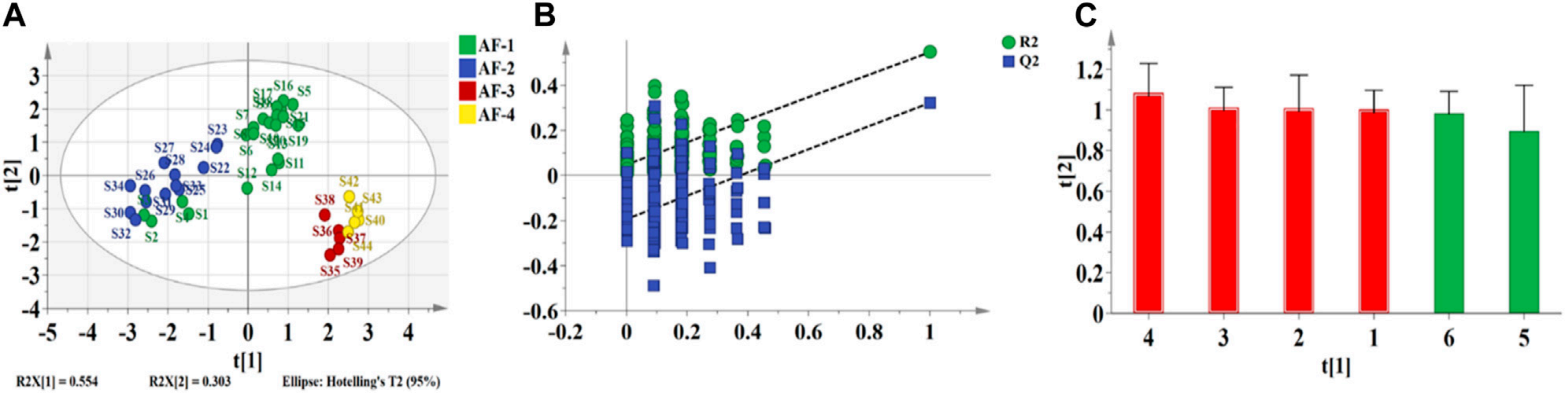
OPLS-DA analysis of Seed. Score plot **(A)** 200X permutation tests **(B)**; VIP** (C)**.

To further investigate the quality differences among *A. fructus* seeds from the four origins, OPLS-DA was performed using the data of six common peaks extracted from the GC fingerprints of the seeds as variables (Figure 4A). The model prediction parameter R^2^Y is the explanation rate of the proposed model for the Y-matrix, and Q^2^ is the predictive power of the model, and the closer their values are to 1 indicates a better fit of the model. The model predicted parameters R^2^Y (0.526) and Q^2^ (0.395) indicated that the model was stable and had some predictive ability. The Yunnan and Guangdong *A. fructus* seeds are in one category and the rest are grouped together (Figure 4A), consistent with the similarity analysis results. To verify whether the model was overfitted, a 200X permutation test was conducted using SIMCA 14.1 (Figure 4B). The model is valid when all Q^2^ and *R* ^2^ values on the left are lower than the original point on the right, or when the blue regression line of Q^2^ intersects the vertical axis (on the left) at or below zero. The intercepts of the model validation parameters *R* ^2^ and Q^2^ were 0.045, and −0.210, respectively. The regression curve of Q^2^ intersected the *Y*-axis below zero, indicating that the model was not overfitted. Subsequently, VIP (Variable Importance for the Projection) analysis was performed, and the larger VIP value indicated that the difference of the peak has a greater impact on the quality of *A. fructus* from different origins. Using a VIP value greater than 1 as the screening criterion, a total of 4 common peaks were screened. The order of influence on sample quality among the peaks was peak 4> peak 3> peak 2 > peak 1.

### 3.3 Bionic sensory analysis

#### 3.3.1 Establishing qualitative models of *Amomi fructus* authenticity and origin with e-tongue

##### 3.3.1.1 Output information of taste sensor

**FIGURE 5:**
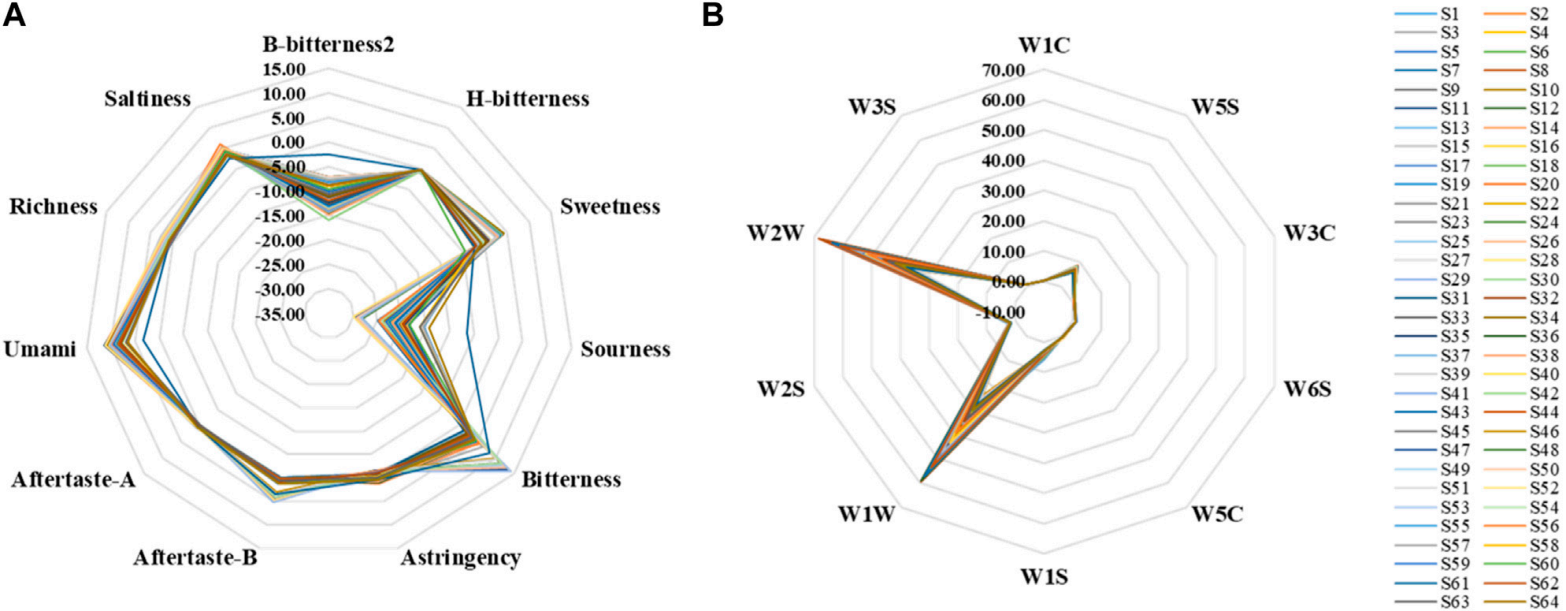
Electronic tongue **(A)**, electronic nose **(B)** output information radar charts.

A total of 11 bionic taste response values were obtained from the measured samples, including B-bitterness2, H-bitterness, Sweetness, Sourness, Bitterness, Astringency, Aftertaste-B, and Aftertaste-A (Figures 5A). The response values of Bitterness, Umami, Richness, and Saltiness for all samples and Sweetness, Aftertaste-A, and Aftertaste-B for some samples were greater than 0. And compared to the rest of the bionic taste response values that were less than 0, these had more practical significance and reference value. The response values of B-bitterness2 and Sourness were significantly higher for S61, which was the only *A. oxyphyllae fructu*s from Guangdong, and lower for Umami compared to the other *A. oxyphyllae fructus* from Guangxi or Hainan. Whether this data is a coincidence or not needs to be further verified by using more samples from Guangdong, and this finding may also provide an idea for the origin identification of *A. oxyphyllae fructus*.

##### 3.3.1.2 Establishing qualitative models of *Amomi fructus* authenticity

**FIGURE 6:**
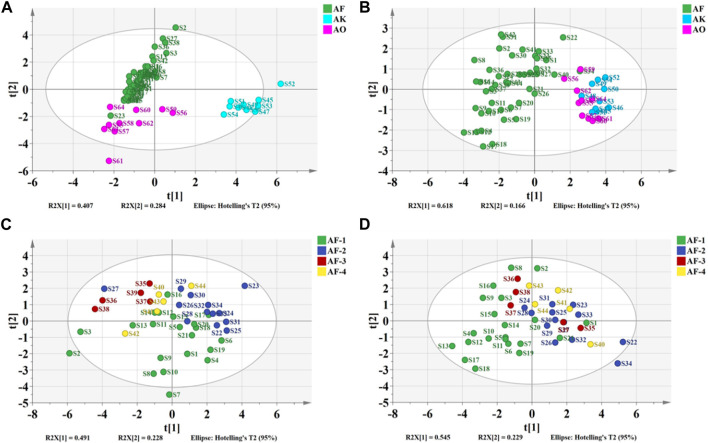
Plots of PCA of the electronic tongue-Authenticity **(A)**, electronic nose-Authenticity **(B)**, electronic tongue-Origin **(C)**, electronic nose-Origin **(D)**.

PCA is an unsupervised data dimensionality reduction method that does not consider sample group information. The dimensionality reduction function of PCA is often used to discover its potential inter-group differentiation trends and to make a basis for subsequent multivariate statistical analysis for classification or prediction (Se et al., 2018). Figure 6A shows a plot of the sample principal component scores created using the taste response values. The first two principal components could explain 69.1% of the variance information of the original data. The two-dimensional plot shows that except for S23, 44 batches of *A. fructus*, 10 batches of *A. katsumadai semen* and 10 batches of *A. oxyphyllae fructus* can be clustered into one category each.

**FIGURE 7:**
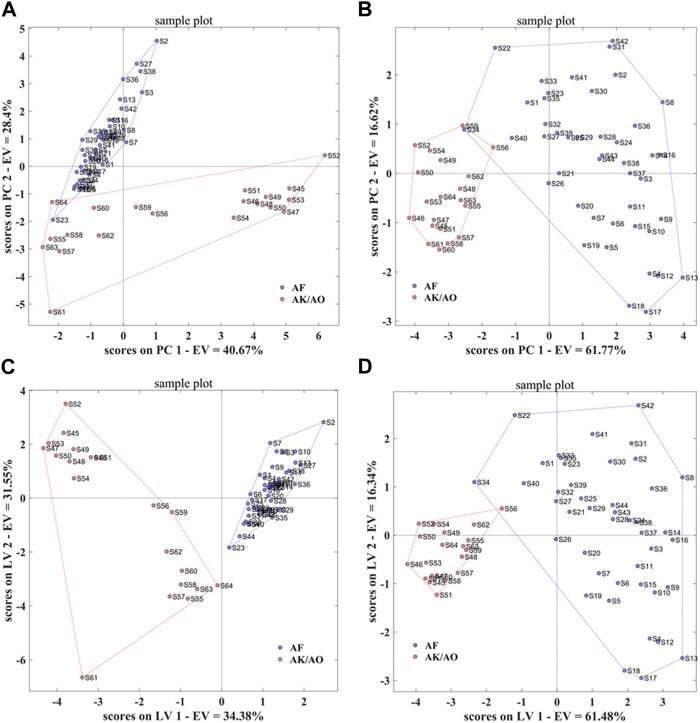
PCA-DA models of the electronic tongue-Authenticity **(A)**, electronic nose-Authenticity **(C)**, PLS-DA models of the electronic tongue-Authenticity **(B)**, electronic nose-Authenticity **(D)**.

Using the taste response values of the above samples as the independent variable *X*) and the species classification of the samples as the dependent variable *Y*), the PCA-DA and PLS-DA authenticity discrimination models of *A. fructus* were established and validated through leave-one-out cross-validation (Figures 7A, B). The *A. fructus* samples are labeled ‘AF’ and the remaining samples as ‘AK/AO’. By preferring the number of principal components to build a qualitative analysis model with better performance of PLS-DA/PCA-DA, the two-dimensional plot shows that the first two principal components can explain 69.07% and 78.39% of the sample information, respectively. The accuracy of both models was 1.00 and there were no assigned samples (Table 2). It shows that the developed model can completely distinguish whether the sample is*Amomi fructus* or not, and the model performs well.

**TABLE 2:** Parameters of each model.

Technology	Category	Model	Training			Cross-validation		
			Error rate	Accuracy	Not assigned	Error rate	Accuracy	Not assigned
E-tongue	authenticity	PCA-DA	0.00	1.00	0.00	0.00	1.00	0.00
		PLS-DA	0.00	1.00	0.00	0.00	1.00	0.00
	origin	PCA-DA	0.17	0.91	0.00	0.21	0.86	0.00
		PLS-DA	0.03	0.94	0.20	0.26	0.75	0.27
E-nose	authenticity	PCA-DA	0.00	1.00	0.00	0.00	1.00	0.00
		PLS-DA	0.00	1.00	0.00	0.00	1.00	0.00
	origin	PCA-DA	0.07	0.75	0.00	0.38	0.75	0.00
		PLS-DA	0.26	0.97	0.25	0.45	0.78	0.18
E-tongue + E-nose	origin	PCA-DA	0.05	0.98	0.00	0.22	0.82	0.00
		PLS-DA	0.00	1.00	0.07	0.23	0.82	0.23

Note: Accuracy is the proportion of correctly classified samples to the participating classified samples. Not assigned samples is the participating modeling samples that cannot be classified.

##### 3.3.1.3 Establishing qualitative models of *Amomi fructus* origin

PCA was performed on the taste response values of 21 batches of *A. fructus* from Yunnan, 13 from Guangdong, 5 from Hainan, and 5 from Myanmar. The first two principal components could explain 71.9% of the variance information of the original data. The results showed that *A. fructus* of each origin could not be clearly clustered into a single category (Figures 6C), indicating that the *A. fructus* taste information across origins was relatively similar, and it was impossible to distinguish them effectively through PCA alone.

**FIGURE 8:**
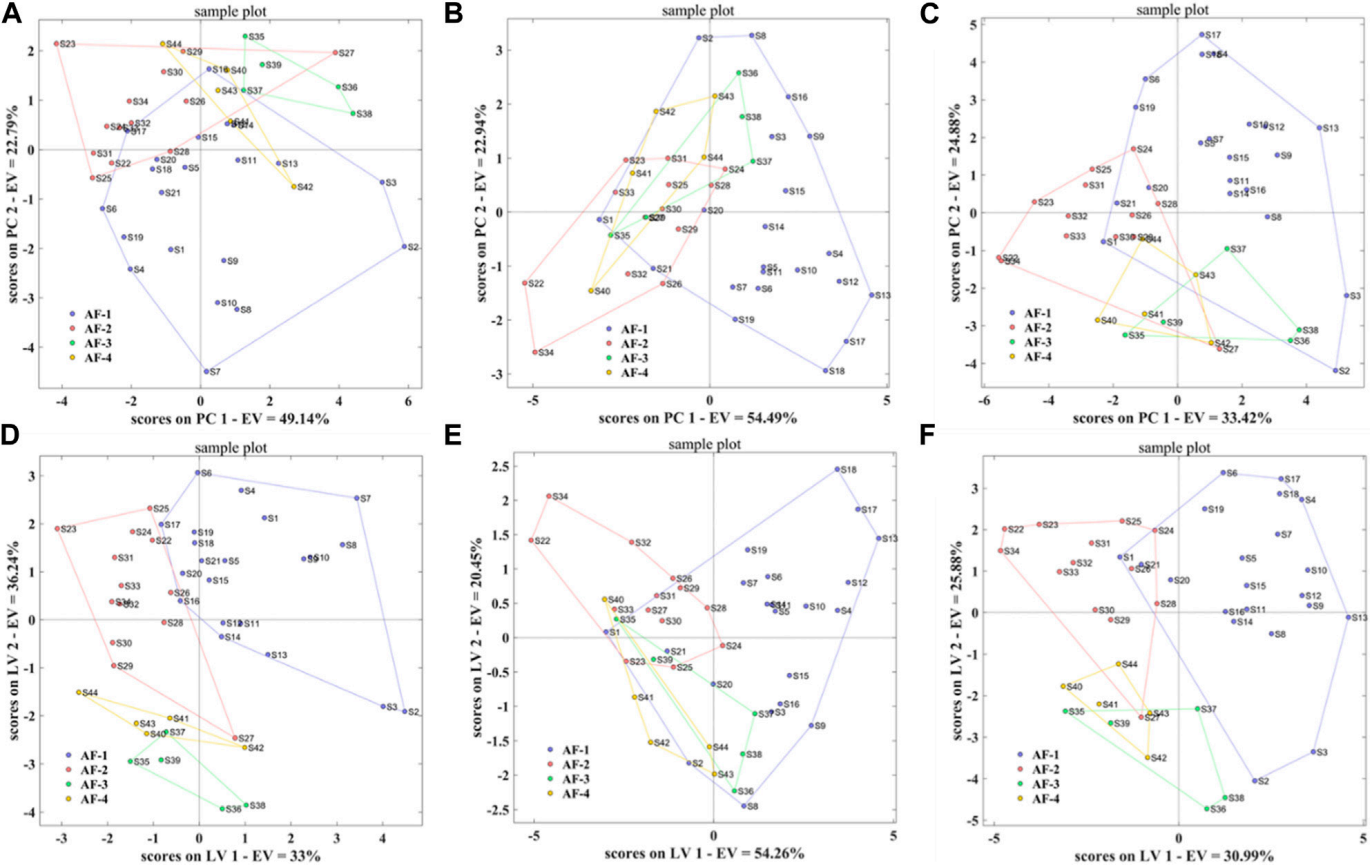
PCA-DA models of the electronic tongue-Origin **(A)**, electronic nose-Origin **(C)** and two-source fusion-Origin **(E)**, PLS-DA models of the electronic tongue-Origin **(B)**, electronic nose-Origin **(D)** and two-source fusion-Origin **(F)**.

Using the taste response values of the above samples as the independent variable *X*) and the origin classification of the samples as the dependent variable *Y*), the PCA-DA and PLS-DA origin discrimination models of the *A. fructus* were established and validated using leave-one-out cross-validation (Figures 8A, B). The *A. fructus* from Yunnan were labeled as ‘AF-1’, from Guangdong as ‘AF-2’, from Hainan as ‘AF-3’, and from Myanmar as ‘AF-4’. The accuracies of the PCA-DA and PLS-DA models were 0.86 and 0.75, respectively, but there were more unassigned samples in the PLS-DA model (Table 2). The performance of the PCA-DA model established by the taste response values was relatively good as it was generally able to accurately distinguish *A. fructus* of different origins.

#### 3.3.2 Establishing the qualitative models of *Amomi fructus* authenticity and origin by e-nose

##### 3.3.2.1 Output information of olfactory sensor

A total of 10 bionic olfactory response values were obtained from the measured samples, including W1C, W5S, W3C, W6S, W5C, W1S, W1W, W2S, W2W, W3S (Figure 5B). The sensor response values for all samples were all greater than 0, and the response values of W1W and W2W were larger, indicating that the samples contained more inorganic sulfur compounds, terpenes, sulfur-containing organic compounds, aromatic compounds, and more organic compounds.

##### 3.3.2.2 Establishing the qualitative models of *Amomi fructus* authenticity

The PCA on the olfactory response values of 44 batches of *A. fructus*, 10 batches of *A. katsumadai semen,* and 10 batches of *A. oxyphyllae fructus* showed that the *A. fructus* were clustered into one category except for S34, and *A. katsumadai semen* and *A. oxyphyllae fructus* were clustered into one category (Figure 6B). The first two principal components could explain 78.4% of the variance information of the original data.

Using the olfactory response values of the above samples as the independent variable *X*) and the species classification of the samples as the dependent variable *Y*), the PCA-DA and PLS-DA authenticity discrimination models of the *A. fructus* were established and validated using leave-one-out cross-validation (Figures 7C, D). The two-dimensional plot shows that the first two principal components can explain 65.93% and 77.82% of the sample information, respectively. The *A. fructus* samples were labeled ‘AF’ and the remaining samples were labeled ‘AK/AO’. The accuracy of both models was 1.00 and there were no unassigned samples (Table 2), indicating that the established models could completely distinguish between *A. fructus* and the others.

##### 3.3.2.3 Establishing qualitative models of *Amomi fructus* origin

The PCA model of the olfactory response values showed that the *A. fructus* of each origin could not be clearly clustered into one category each. This indicated that the *A. fructus* olfactory information, as with the taste information, was similar across origins so their origins could not be effectively distinguished by unsupervised PCA alone.

Using the olfactory response values of the above samples as the independent variable *X*), and the origin classification of the samples as the dependent variable *Y*), the PCA-DA and PLS-DA origin discrimination models of the *A. fructus* were established and validated using leave-one-out cross-validation (Figures 8C, D). The accuracies of the PCA-DA and PLS-DA models were 0.75 and 0.78, respectively, but there were more unassigned samples in the PLS-DA model (Table 2). The PCA-DA model established by the olfactory response values was generally able to accurately distinguish the *A. fructus* of different origins.

#### 3.3.3 Establishing qualitative models of *Amomi fructus* origin using e-tongue and e-nose

The accuracy of the qualitative models of *A. fructus* authenticity established by a single type of bionic sensory data all reached 1.00, while the accuracy of the qualitative origin models was relatively low. In order to improve data utilization and model performance, the PCA-DA and PLS-DA origin identification models were established by fusing the above two types of bionic sensory data, taking the fused data as the independent variable *X*) and the *A. fructus* origin classification as the dependent variable *Y*). The accuracy of the PCA-DA and PLS-DA models was 0.82 (Figures 8E, F), and there were still unassigned samples in the PLS-DA model. Compared to the qualitative origin model established by bionic taste data, the accuracy of the PCA-DA model was reduced, while the number of unassigned samples in the PLS-DA model was reduced. Compared with the qualitative origin model established by bionic olfactory data, the accuracy of both types of models was improved, while the number of unassigned samples in the PLS-DA model was increased.

#### 3.3.4 Establishing quantitative models of Amomi fructus using volatile component content and bionic sensory data

**FIGURE 9:**
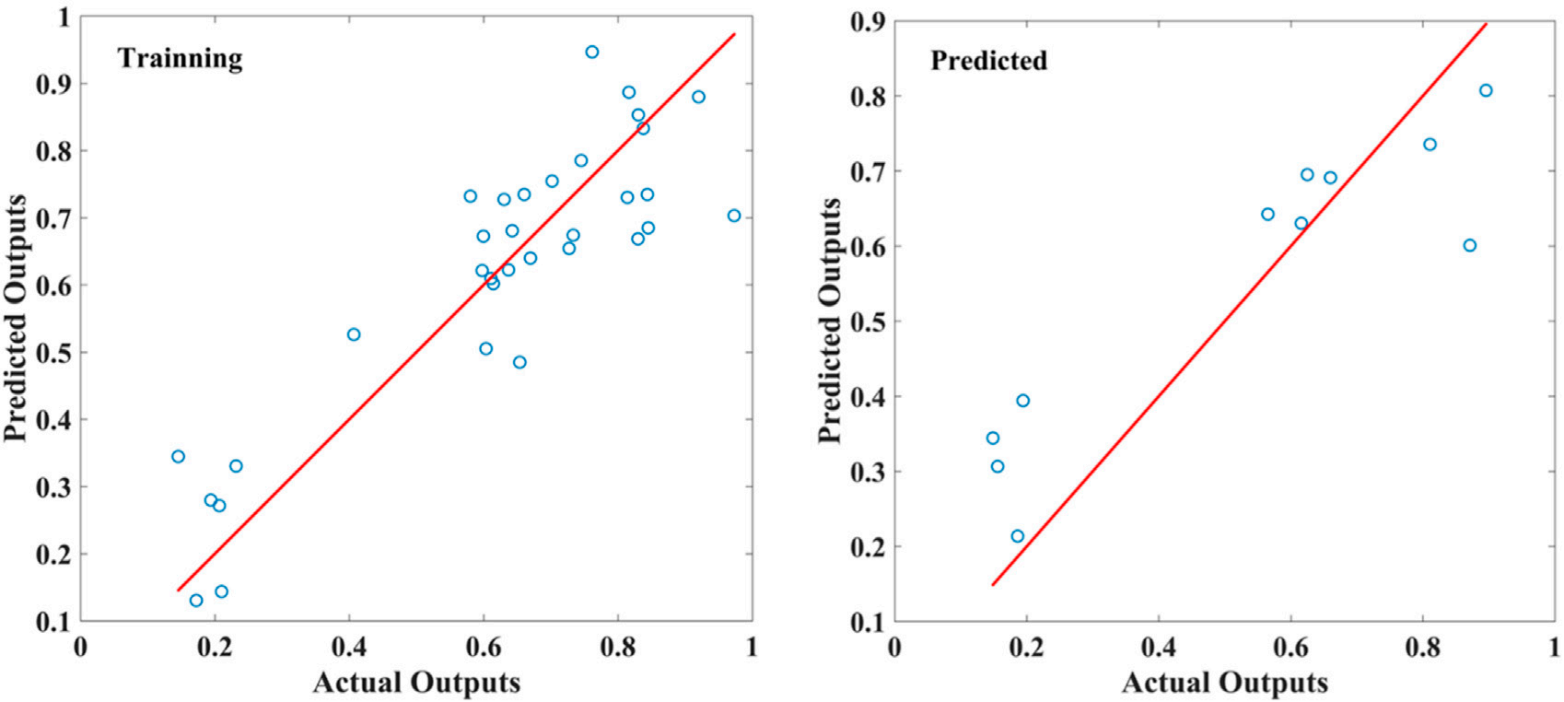
Predicted and actual values of PLSR models for two-source fusion + bronyl acetate.

A PLSR analysis of *A. fructus* was carried out with the fusion of two types of bionic sensory data as the independent variable *X*), and the contents of camphor, borneol, and bornyl acetate in *A. fructus* as the dependent variable *Y*). Samples were divided into a training set of 33 and a test set of 11. With an *R* ^2^ of 0.7914, the fusion model outperformed the PLSR models established by a single type of bionic sensory data (Figure 9). The parameters of PLSR models are reported in Table 3. This result illustrated the strong correlation between borneol acetate and bionic sensory data. This model can be used to evaluate the quality of *A. fructus* and highlights the importance of borneol acetate in the variety and quality evaluation of *A. fructus*.

**TABLE 3:** Parameters of PLSR models.

Model	Optlv	R-Square	RMSEF	RESRP
E-tongue + Camphor	4	0.7218	0.0585	0.0694
E-tongue + Borneol	1	0.2283	0.0207	0.0107
E-tongue + Bronyl acetate	1	0.4606	0.1732	0.1684
E-nose + Camphor	6	0.4993	0.0761	0.1362
E-nose + Borneol	5	0.3424	0.0181	0.0173
E-nose + Bronyl acetate	6	0.6470	0.1392	0.2809
Two-source fusion + Camphor	2	0.6901	0.0653	0.0658
Two-source fusion + Borneol	2	0.2973	0.0195	0.0140
Two-source fusion + Bronyl acetate	5	0.7914	0.1050	0.1349

Note: Optlv is the number of latent variables that reach the minimum RMSECV, by ten-fold interaction validation. R-Square is the coefficient of determination, and the closer R-Square is to 1 the better the model fit. RMSEF, is the root mean squared error of the training set; RESRP, is the root mean squared error of the prediction set.

## 4 Conclusion

The qualitative models of *A. fructus* authenticity were the optimal model with an accuracy of 1.00. The qualitative origin model using the PCA-DA established by the e-tongue was optimal, with an accuracy of 0.86. The PLSR model established by two types of sensory fusion data combined with bornyl acetate content was optimal, with an *R* ^2^ of 0.7914. Our study reveals that the use of e-tongue and e-nose combined with GC can be used to evaluate the variety and quality of *A. fructus* quickly and accurately, and the introduction of MIF technology can improve the prediction accuracy of the model to some extent. This study provides a potential tool for the rapid and accurate evaluation of the variety and quality of *A. fructus* and also provides a promising method for evaluating the variety and quality of other traditional Chinese medicines and foods.

## Data Availability

The original contributions presented in the study are included in the article/Supplementary Material, further inquiries can be directed to the corresponding authors.
